# Late-Onset Carnitine–Acylcarnitine Translocase Deficiency With *SLC25A20* c.199-10T>G Variation: Case Report and Pathologic Analysis of Liver Biopsy

**DOI:** 10.3389/fped.2020.585646

**Published:** 2020-10-30

**Authors:** Min Chen, Yao Cai, Sitao Li, Hui Xiong, Mengxian Liu, Fei Ma, Xin Xiao, Hu Hao

**Affiliations:** Department of Pediatrics, The Sixth Affiliated Hospital of Sun Yat-sen University, Guangzhou, China

**Keywords:** carnitine-acylcarnitine translocase deficiency, late-onset CACTD, *SLC25A20* gene, liver biopsy, steatosis, iron deposition

## Abstract

**Introduction:** Carnitine–acylcarnitine translocase deficiency (CACTD) is a rare and life-threatening autosomal recessive disorder of mitochondrial fatty acid oxidation caused by variation of the Solute carrier family 25 member 20 (*SLC25A20*) gene. Carnitine–acylcarnitine translocase is one of the crucial transport proteins in the oxidation process of mitochondrial fatty acids. In Asia, the c.199-10T>G splice site variation is the most frequently reported variant of *SLC25A20*. Patients with CACTD with c.199-10T>G variation usually present with a severe clinical phenotype.

**Materials and Methods:** Herein, we report a neonatal case of late-onset CACTD in mainland China. Symptoms emerged 61 days after birth; the patient presented with a severe metabolic crisis, and her clinical condition rapidly deteriorated, and she died of respiratory insufficiency and cardiac arrest at 61 days. We present the clinical and biochemical features of this patient and briefly review previously reported CACTD cases with c.199-10T>G variation.

**Results:** Acylcarnitine profiling by tandem mass spectrometry and high-throughput sequencing revealed that our patient was homozygous for the c.199-10T>G variation, confirming the diagnosis of CACTD. Histopathologic analysis of the liver by Prussian blue staining showed focal iron deposition in hepatocytes, and electron microscopy analysis revealed a large number of lipid droplet vacuoles in diffusely distributed hepatocytes.

**Conclusion:** The development of CACTD in our patient 61 days after birth is the latest reported onset for CACTD with *SLC25A20* c.199-10T>G variation. Early recognition of symptoms and timely and appropriate treatment are critical for improving the outcome of this highly lethal disorder. Death from late-onset CACTD may be caused by the accumulation of long-chain fatty acids as well as iron deposition in the heart leading to heart failure.

## Introduction

Carnitine–acylcarnitine translocase deficiency (CACTD) (Online Mendelian Inheritance in Man #212138) is a rare and life-threatening autosomal recessive disorder of mitochondrial fatty acid oxidation (FAO) resulting from variation of the Solute carrier family 25 member 20 (*SLC25A20*) gene. Carnitine–acylcarnitine translocase (CACT) is one of the crucial transport proteins in the oxidation process of mitochondrial fatty acids. It mainly catalyzes the exchange of acylcarnitine and free carnitine on both sides of the mitochondrial inner membrane and plays an important role in the transport of long-chain (LC) acylcarnitine into mitochondria. The classic phenotype of CACTD includes neonatal hypoketotic hypoglycemia, hyperammonemia, cardiomyopathy, hepatopathy, and myopathy (1). The estimated incidence of CACTD is 1/60,000 in Hong Kong (2) and 1/76,894 in Hunan, China (3). The majority of reported CACTD cases have resulted in unexplained sudden death during the neonatal period (4, 5).

The *SLC25A20* gene is located on 3p21.31 and contains nine exons (6). To date, at least 42 different pathogenic or possibly pathogenic variants of *SLC25A20* have been identified that cause CACTD (Human Genome Mutation Database [HGMD] Professional 2018.4), including 20 missense or nonsense variations, 10 small deletions, 2 small insertions, 1 small indel, 4 gross deletions, and 5 splice site variations. In Asia, the c.199-10T>G splice site variation is the most frequently reported variant; patients harboring this variation typically present with a severe clinical phenotype (3).

In most cases, the c.199-10T>G variation affects children within a few days after birth. Here, we present the case of a female patient who developed symptoms of the disease 61 days after birth, which is the latest reported manifestation for this variant of *SLC25A20*. We describe the clinical, biochemical, histopathologic, and molecular characteristics of this patient and briefly review other cases of CACTD with the *SLC25A20* c.199-10T>G variation that have been reported in the literature.

## Materials and Methods

### Case Description

The female infant was the fourth fetus and third delivery for the mother and was born by cesarean section at 36 weeks gestation, with a birth weight of 2.2 kg. Her amniotic fluid was clean at birth, and she was breastfed after birth. The father was healthy, and the mother was a carrier of chronic hepatitis B virus, and they were not close relatives. The mother's first child was aborted for personal reasons; the second child, a boy, died on the day of birth of an unknown cause. The third child, a 5-year-old girl, is in good health. The family pedigree is shown in **Figure 4**. After birth, the child was diagnosed as a premature infant and hospitalized for 13 days. When discharged from the hospital, the child's general condition was good, and she showed good feeding behavior and reflexes. However, weight gain was not ideal, and the child's body mass was only 3.66 kg at 2 months. At 61 days, she was admitted to another hospital for 1 day due to fever; her responses deteriorated, and she was transferred to our hospital for further treatment. A physical examination conducted upon admission revealed poor response, lethargy, shortness of breath, and low muscle strength and tone in the extremities. Routine laboratory tests showed that the patient had hypoglycemia (glucose: 0.28 mmol/L) and elevated myocardial enzymes [creatine kinase (CK): 569 U/L; CK-MB: 136 U/L] and transaminases (alanine aminotransferase: 169 U/L; aspartate aminotransferase: 239 U/L). Even after symptomatic treatments such as increasing blood sugar, nourishment of the heart, and protection of the liver, the patient's condition gradually deteriorated. Five hours after admission, apnea and cardiac arrest occurred, accompanied by low peripheral capillary oxygen saturation (70%); several resuscitation attempts failed, and the patient died.

Heel blood and urine samples were collected for tandem mass spectrometry (MS/MS) and gas chromatography–mass spectrometry analyses. All mass spectrometry experiments were performed on a Waters TQD mass spectrometer (Waters, Milford, Massachusetts, USA). Data were acquired and analyzed with Waters MassLynx v4.1 software. The urine samples were treated with urea removal, internal standard add, protein removal, vacuum drying, and trimethylsilyl derivatization and then analyzed by JMS-Q1000GC GC-MS (JEOL, Japan) for such as organic acids, amino acids, carbohydrate, polyols, purines, and pyrimidines in the urine. After the child died, we immediately biopsied her liver with the consent of her parents. Liver tissues were fixed in 4% paraformaldehyde, embedded in paraffin, and sectioned at 5 μm. Hematoxylin, eosin, Prussian blue, reticular fiber, masson, and picrosirius red staining were performed according to standard procedures.

### Genetic Testing

This study was approved by the ethics committee of the Sixth Affiliated Hospital of Sun Yat-sen University (approval no. 2017ZSLYEC-105) and carried out after obtaining informed consent from the parents of the patient. Peripheral venous blood samples were obtained from the patient and her parents in 3-ml ethylenediaminetetraacetic acid anticoagulant tubes, and genomic DNA was extracted using the Solpure Blood DNA kit (Magen Biotechnology, Guangzhou, China). After DNA fragmentation, end-repair, amplification, and purification, the genomic library was established. Target genes were captured with the Clinical 4000 Pathogenic Gene Package (Guangzhou Jia-Jian Medical Testing, Guangzhou, China). The detection interval of the gene package for this syndrome included 4,047 related genes, and the 55,698 coding regions contained a total of 11,781,176 bases with an average coverage depth of 227 ± 143x, accounting for 99.6% of the coverage interval >10× and 99.5% of the coverage interval >20×. High-throughput sequencing was performed on a Nextseq 500 sequencing platform (Illumina, San Diego, CA, USA). Bioinformatics methods analyzed the sequencing results, and Sanger sequencing was performed to verify the positive loci of the patient, her parents, and her older sister. The reference human genome was hg19 (February 2009; University of California at Santa Cruz, Santa Cruz, CA, USA), and the data were interpreted according to the American College of Medical Genetics and Genomics guidelines.

## Results

### Tandem Mass Spectrometry and Gas Chromatography–Mass Spectrometry

At the time of symptom emergence, the results of the MS/MS analysis revealed elevated levels of Gln, His, Met, Orn, C16, C22, and C24 and reduced levels of Glu, C0, C2, C3, C4-OH, C5, and C8 in the blood (Table 1). Gas chromatography–mass spectrometry analysis of the urine sample showed increased concentrations of several amino acids, 4-hydroxyphenyllactic acid, and pyrimidines.

**Table 1:** Tandem mass spectrometry results at the onset of carnitine–acylcarnitine translocase deficiency.

**Species**	**Concentration (μmol/L)**	**Reference range**
Gln	26.8	2.0–20.0
His	246.3	10.0–200.0
Met	42.8	8.0–38.0
Orn	86.4	5.0–50.0
Glu	55	60.0–200.0
C0	3.3	14.0–55.0
C2	2.6	6.0–30.0
C3	0.19	0.30–3.00
C4-OH	0.02	0.03–0.18
C5	0.05	0.06–0.30
C8	0.02	0.03–0.30
C16	4.3	0.5–2.5
C18	0.85	0.20-1.40
C20	0.12	0.02-0.12
C22	0.18	0.03–0.16
C24	0.27	0.02–0.12

### Liver Pathology

**Figure 1:**
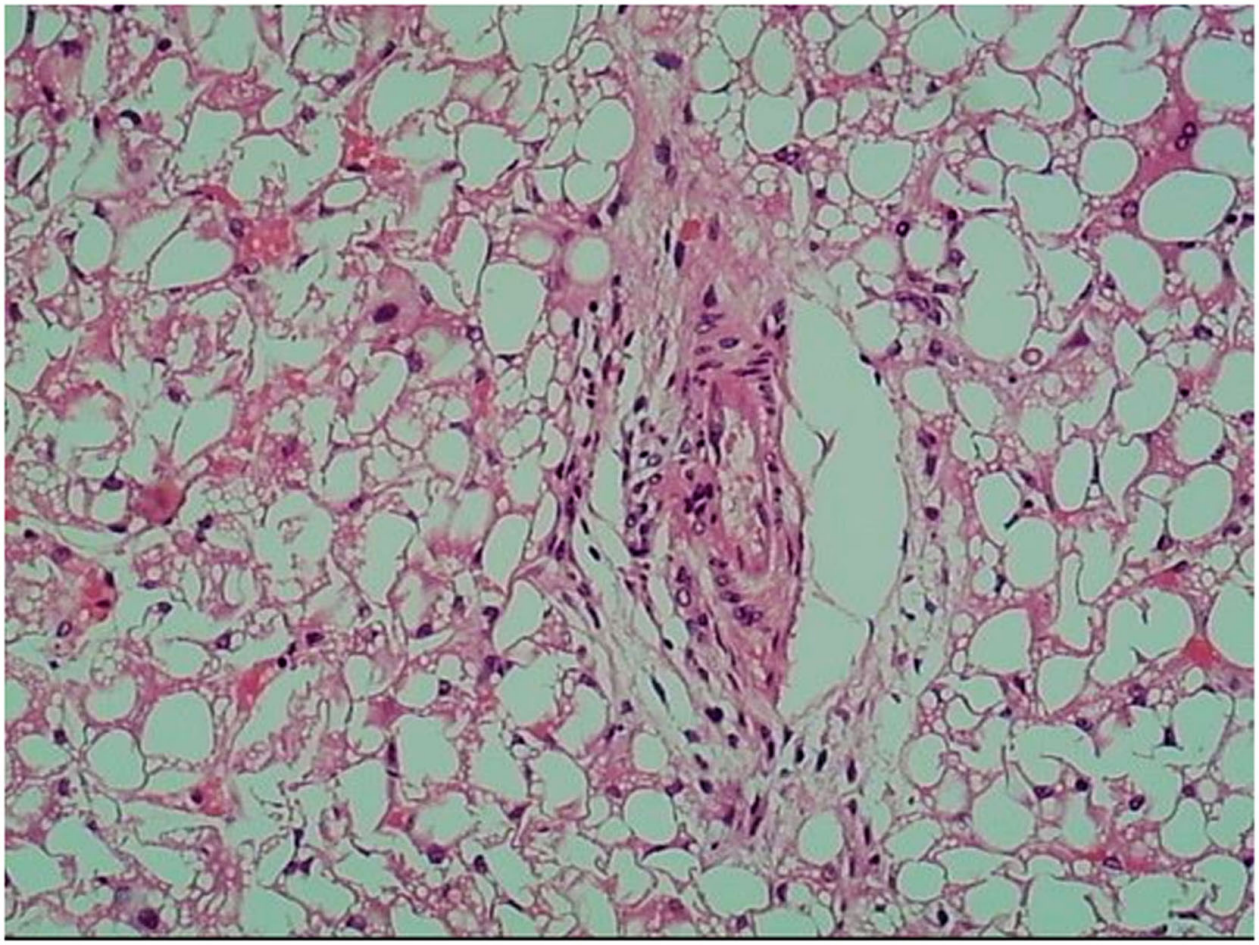
Hematoxylin and eosin staining of liver tissue from patient with CACTD. Extensive vacuolar degeneration was observed (100× magnification).

**Figure 2:**
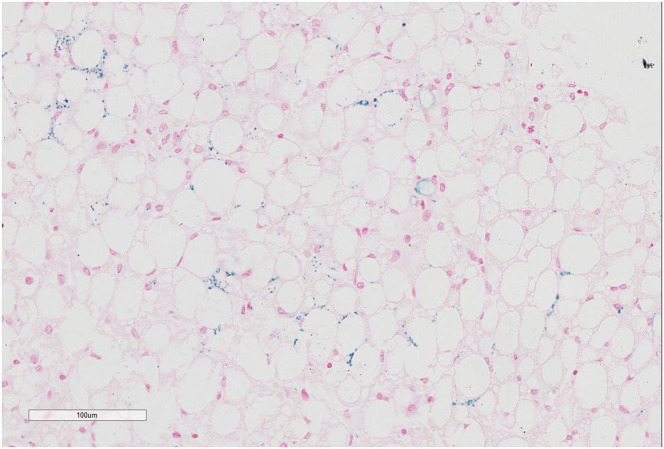
Prussian blue staining of liver tissue from patient with CACTD. Iron deposition was visible in liver cells (100× magnification).

**Figure 3:**
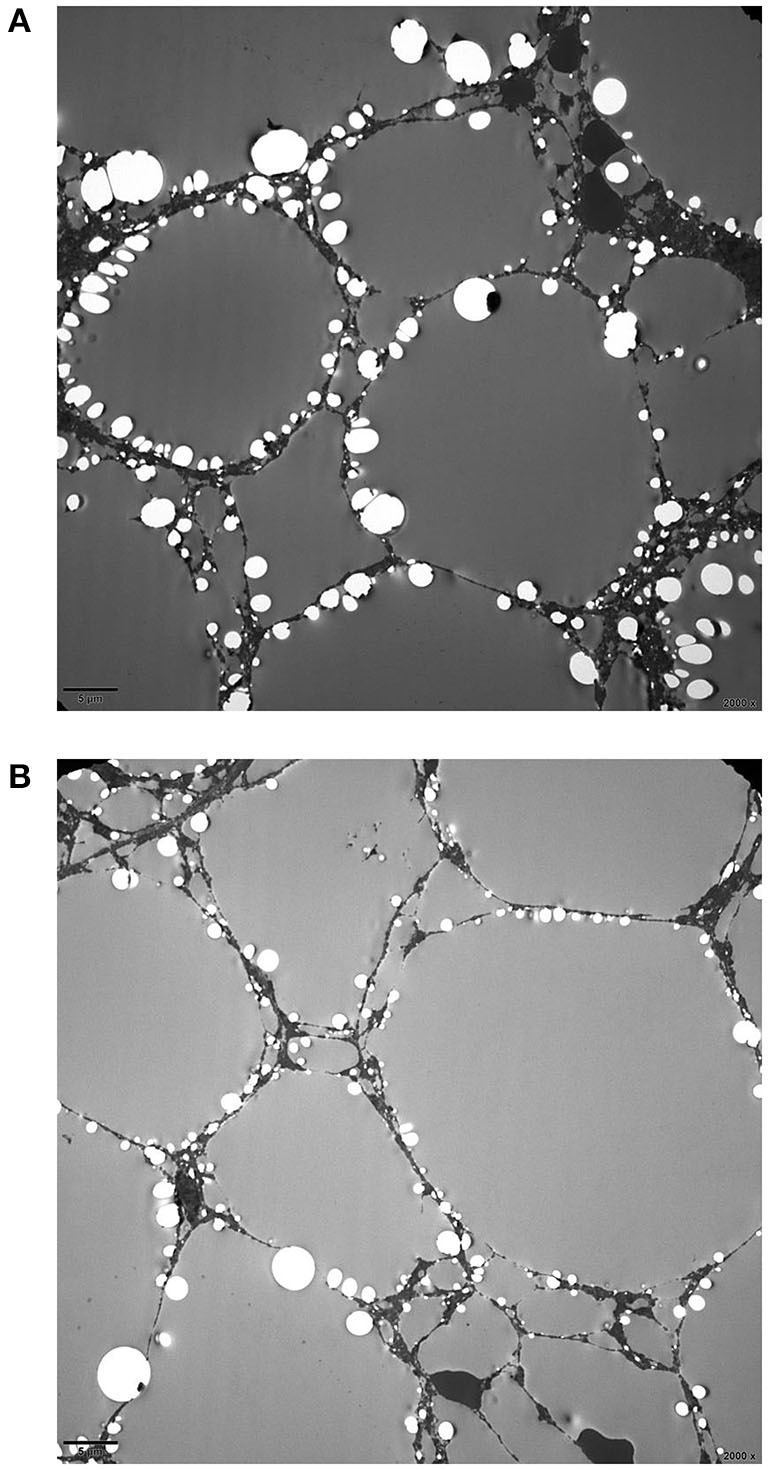
Electron micrograph of the liver from patient with CACTD. **(A,B)** A large number of lipid droplet vacuoles were observed in diffusely distributed hepatocytes (2,000× magnification).

Prussian blue staining showed focal iron deposition in hepatocytes, and examination of liver tissue by electron microscopy revealed many lipid droplet vacuoles in diffusely distributed hepatocytes containing round, square, and irregularly shaped crystals, consistent with fatty liver disease (Figures 1–3). Masson and Picrosirius red staining showed no proliferation of fibrous tissue; reticular fiber staining showed the preservation of the reticular scaffold of hepatocytes.

### Results of Gene Sequencing

**Figure 4:**
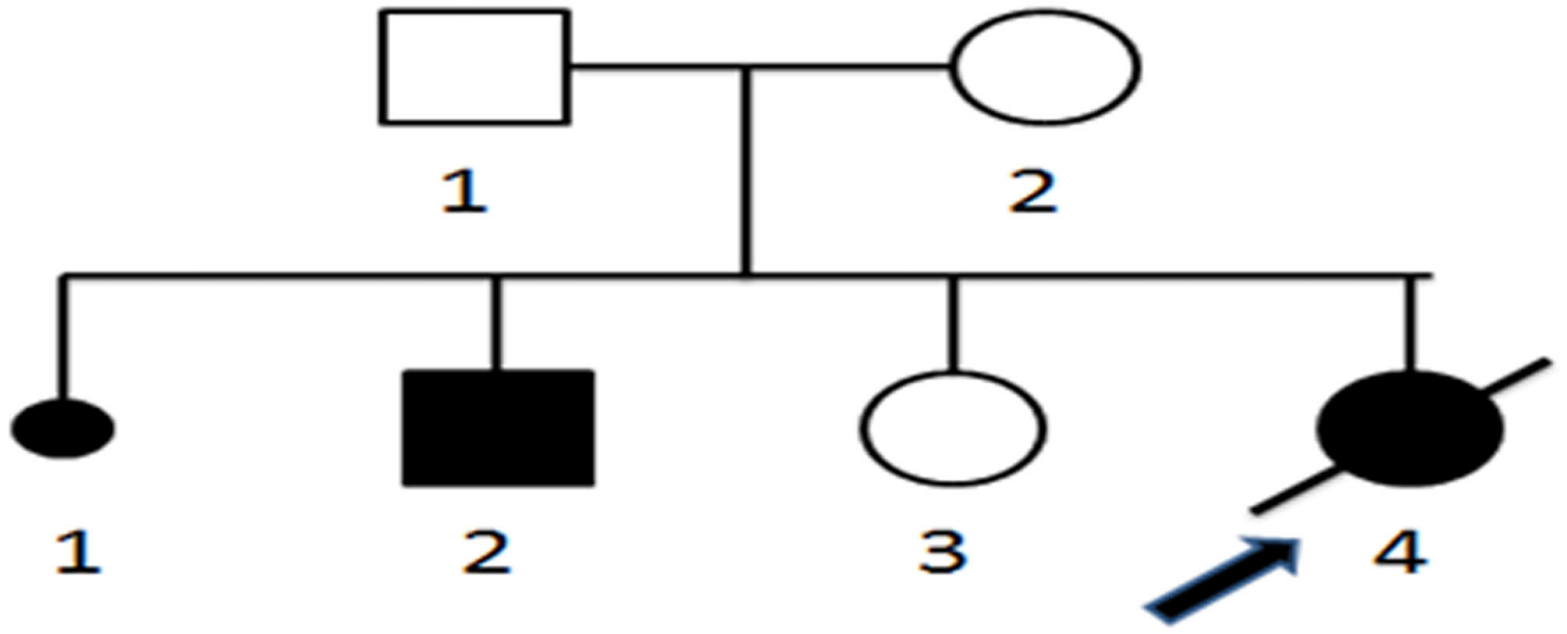
Pedigree of neonatal sudden death carnitine–acylcarnitine translocase deficiency. 1–4, Order of family members; □ normal male; ◦, normal female; ■ male patient; •, female patient; 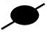, patient who died of CACTD; 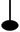, stillbirth or abortion; 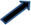, proband.

**Figure 5:**
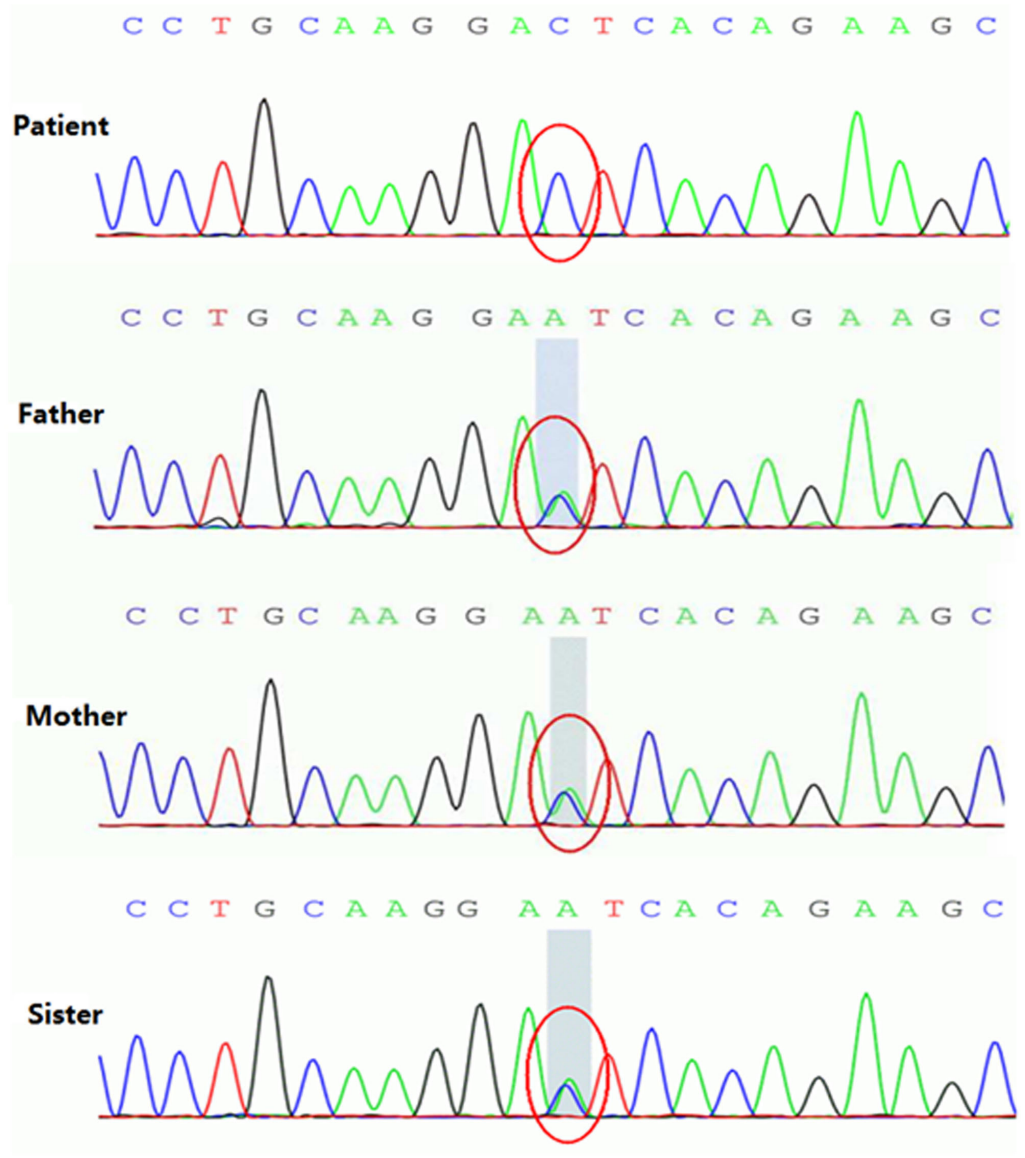
Homozygous variant of the c.199-10T>G splice site in the *SLC25A20* gene of patient with CACTD. Both parents and older sister were heterozygous carriers of the mutation.

A homozygous c.199-10T>G splice site variation was detected in the *SLC25A20* gene of the patient by high-throughput sequencing. Combined with the clinical manifestations, the child was diagnosed with CACTD. Both parents were heterozygous carriers of the variation and had no clinical symptoms. The c.199-10T>G variation was confirmed in all subjects by Sanger sequencing, which showed that the older sister of the patient was also a carrier (Figures 4, 5).

### Literature Search Results

Using “Carnitine-acylcarnitine translocase deficiency,” “CACTD,” “*SLC25A20* gene,” and “c.199-10T>G” as keywords, we searched the Online Mendelian Inheritance in Man database and PubMed HGMD up to May 2020 and found 12 reports on CACTD with the c.199-10T>G variation. The clinical features and prognosis of the 25 patients are shown in Table 2. Most of the patients developed symptoms within 3 days after birth. The most common symptoms were hypoglycemia and cardiac arrest. The case fatality rate was 96% (25/26). Clinical symptoms in our patient appeared at 61 days, which is the latest onset that has been reported to date for this variant of *SLC25A20*. The disease is mainly manifested as abnormal liver function and sudden cardiac arrest. The severe steatosis observed by histopathologic analysis of the liver suggests that our patient may have had an abnormal liver function before 61 days, masked by compensatory mechanisms. As the patient did not show obvious hypoglycemia, jaundice, or feeding intolerance, her parents did not bring her to the hospital for examination; therefore, the specific disease onset time could not be determined. One study examining the effect of the *SLC25A20* c.199-10T>G variation on messenger RNA products found that it disrupted the second and third transmembrane domains of the protein, resulting in loss of translocase activity (17), which could explain the severe phenotype and high mortality associated with this variant.

**Table 2:** Characteristics of patients with carnitine–acylcarnitine translocase deficiency with c.199-10T>G mutation.

**Patient**	**Sex**	**Country**	**Gene variant**	**Age of onset**	**Symptom**	**Outcome**	**References**
1	F	China	c.199-10T>G Homozygous variant	61 days	Hepatic dysfunction and hypoglycemia	Died of respiratory insufficiency and cardiac arrest 61 days after birth	This report
2	M	China	c.199-10T>G+c.120delT Heterozygous variant	36 h	Seizures and respiratory insufficiency	Died of respiratory failure at 37 months	Stanley et al. (7)
3	F	China	c.199-10T>G+c.326delG Heterozygous variant	27 h	Lethargy, feeding difficulties	Died 31 h after birth	Chalmers et al. (8)
4	F	Vietnam	c.199-10T>G Homozygous variant	2 days	Hypoglycemia	Died of respiratory arrest 6 months after birth	Hammond et al. (9) Costa et al. (10)
5	M	Vietnam	c.199-10T>G Homozygous variant	Within 3 days	Hypoglycemia and hypopnea	Sudden death (unknown time)	Costa et al. (10)
6	M	Vietnam	c.199-10T>G Homozygous variant	Within 3 days	Unknown	Sudden death 2 months after birth	Costa et al. (10)
7	M	Japan	c.199-10T>G+c.576G>A Heterozygous variant	2 days	Respiratory insufficiency	Died at 33 months after birth	Fukushima et al. (11)
8	M	Hong Kong, China	c.199-10T>G Homozygous variant	41 h	Cardiac arrest	Died of cardiac arrest 3 days after birth	Lam et al. (12) Lee et al. (13)
9	F	Hong Kong, China	c.199-10T>G Homozygous variant	32 h	Cardiac arrest	Still alive and followed up for 32 months after birth	Lee et al. (13)
10	M	Hong Kong, China	c.199-10T>G Homozygous variant	28 h	Respiratory insufficiency and cardiomyopathy	Died of cardiac arrest 38 h after birth	Lee et al. (13)
11	M	Thailand	c.199-10T>G Homozygous variant	10 h	Hypothermia followed by cardiac arrest 60 h after birth	Died of upper gastrointestinal bleeding and metabolic disorders at the age of 2 years and 8 months	Vatanavicharn et al. (6)
12	F	Thailand	c.199-10T>G Homozygous variant	2 days	Lethargy, difficulty feeding, and cardiac arrest	Died of cardiac arrest 4 months after birth	Vatanavicharn et al. (6)
13	M	China	c.199-10T>G Homozygous variant	25 min	Hypoglycemia, apnea, and seizures	Died of cardiac arrest 78 h after birth	Yan et al. (3)
14	F	China	c.199-10T>G+C.1A>G	52 h	Hypoglycemia and hypotension	Died of heart failure 6 days after birth	Yan et al. (3)
15	M	China	c.199-10T>G Homozygous variant	2 days	Hypoglycemia	Died of heart failure 3 days after birth	Fan et al. (14)
16	M	China	c.199-10T>G Homozygous variant	1.5 days	Hypoglycemia, seizures, and apnea	Died of heart failure 2 days after birth	Fan et al. (14)
17	M	China	c.199-10T>G Homozygous variant	3 days	Hypoglycemia	Sudden death 4 days after birth	Fan et al. (14)
18	F	China	c.199-10T>G Homozygous variant	30 days	Hypoglycemia	Sudden death 30 days after birth	Fan et al. (14)
19	M	China	c.199-10T>G/c.719-8_c.719-1dupCCCCACAG	1 days	Hypoglycemia	Died 3 days after birth from cardiogenic shock with malignant ventricular arrhythmia and pulmonary hemorrhage	Fan et al. (14)
20	F	China	c.199-10T>G/c.719-8_c.719-1dupCCCCACAG	1 day	Hypoglycemia	Died of cardiac arrest 8 days after birth	Tang et al. (15)
21	M	China	c.199-10T>G Homozygous variant	3 days	Hypoglycemia	Sudden death 2 months after birth	Tang et al. (15)
22	F	China	c.199-10T>G Homozygous variant	2 days	Lethargy	Died of cardiac arrest 3 days after birth	Tang et al. (15)
23	M	China	c.199-10T>G Homozygous variant	2 days	Lethargy and hypotonia	Died of respiratory distress and arrhythmia	Tang et al. (15)
24	F	China	c.199-10T>G Homozygous variant	2 days	Lethargy and hypoglycemia	Died of cardiac arrest 3 days after birth	Tang et al. (15)
25	M	China	c.199-10T>G Homozygous variant	2 days	Hypoglycemia, lethargy, and hypotonia	Died of heart failure 4 days after birth	Liu et al. (16)
26	M	China	c.199-10T>G Homozygous variant	3 days	Lethargy and cyanosis	Died of arrhythmia and heart failure 3 days after birth	Liu et al. (16)

*F, female; M, male*.

## Discussion

CACTD is a rare autosomal recessive genetic disease characterized by LC FAO disorder. Five cases of CACTD were identified in ~500,000 newborns in Guangzhou, China, between 2016 and 2017, for an estimated incidence of at least 1:100,000 (18). The first case of CACTD was described in 1992, and only 55 cases have been reported worldwide in the two decades since (3). The first three cases of CACTD in the Chinese population were reported in Hong Kong (13). Two more cases were identified in 153,789 newborns screened over a 3-year period from 2015 to 2017 in Hunan province, China (3). To date, there have been 20 cases of CACTD in China, all with the c.199-10T>G variation. Children usually develop symptoms after a long period of starvation or infection; patients who develop symptoms in the neonatal period have a high fatality rate, whereas those who develop symptoms, later on have a good prognosis (19). In most reported cases, patients developed symptoms in the neonatal period, and their condition rapidly deteriorated, resulting in death; this was especially true in cases with the c.199-10T>G variation. In our patient, the disease developed slowly and manifested as sudden hypoglycemia, respiratory insufficiency, and cardiac arrest before death.

The causative gene of CACTD is *SLC25A20*, which is located on chromosome 3p21.31 and contains nine exons encoding 301 amino acids. To date, at least 42 different pathogenic or possibly pathogenic variations have been identified for CACTD (HGMD Professional 2018.4). The most common are the c.199-10T>G splice site variation, which is observed in patients from East Asia (Japan, China, and Vietnam), and the c.713A>G missense variation, which has been detected in patients of Middle Eastern origin. The c.199-10T>G variation is located in a protective lasso branching sequence of *SLC25A20* intron 2 (6); according to the Exome Aggregation Consortium, it is a common hot spot variation in Asian populations that is detected at a frequency of 0.4‰. However, one study identified 11 carriers in sequencing data of 2,184 people in Guangxi Zhuang Autonomous Region, corresponding to a frequency of 5‰ (14), which is much higher than that in the Exome Aggregation Consortium database.

Routine biochemical tests of CACTD patients typically reveal hypoglycemia and elevated CK and liver enzymes, with a lack of specific diagnostic indicators. Early detection of the disease is mainly through neonatal genetic screening for metabolic diseases. The MS/MS results showed that extremely LC acylcarnitine (i.e., C16, C18, C16:1, C16:1-OH, C18:2, C18:1) accompanied by decreased or normal levels of C0 are indicative of CACTD. However, as the clinical manifestations of severe neonatal and infant carnitine palmityl transferase deficiency are similar to those of CACTD—including acylcarnitine changes in the MS/MS profile—genetic testing or enzymatic analysis is required for definitive diagnosis.

The main principles underlying the treatment of children with CACTD are to avoid malnutrition, prevent infection, and adhere to a high-carbohydrate/low-fat diet (18, 20). During the acute onset of CACTD, continuous high-speed intravenous infusion of glucose solution should be carried out in addition to the reduction of blood ammonia and the administration of other symptomatic and supportive treatments. Long-term treatment should be based on diet control, with supplementation of essential amino acids and fatty acids and the restriction of long-chain fatty acid (LCFA) intake. Screening for neonatal genetic and metabolic diseases can detect CACTD early on; however, although early treatment can improve survival, the prognosis of most children is extremely poor. Therefore, for carriers of an identified variation, a prenatal diagnosis should be performed when pregnancy is confirmed to prevent the birth of a fetus with the variation, which is essential for preventing and reducing the incidence of CACTD.

Our patient harbored the homozygous c.199-10T>G variation of the *SLC25A20* gene, which generates a truncated protein, causing severe clinical phenotypes, including neonatal death (15). Mitochondrial FAO is a major source of energy during prolonged fasting and cardiac and skeletal muscle during long-term exercise (21). The carnitine cycle transfers LCFAs such as acylcarnitine from the cytosol into the intramitochondrial space where mitochondrial FAO occurs (1, 22). When fat is broken down under conditions of starvation, CACT deficiency can lead to the accumulation of toxic LC acylcarnitine in the heart, liver, or skeletal muscle, leading to heart failure, arrhythmia, and cardiac arrest (3). Newborns lacking CACT are particularly vulnerable in the first few postnatal days because of their low oral intake of nutrients and low glycogen reserves. Our patient survived for 2 months after birth, possibly because of frequent breastfeeding by her mother. Nonetheless, toxic LC acylcarnitine slowly accumulated in the patient's liver and heart, and the postmortem examination revealed iron deposition and severe steatosis in the liver.

Chronic liver disease can lead to iron metabolism disorder and excessive iron deposition in the body (23), which is associated with fatal complications such as cirrhosis and heart failure (24–26). Death from late-onset CACTD may be caused not only by the accumulation of LCFAs but also by iron deposits that lead to heart failure. We did not measure serum iron and ferritin levels or perform a histopathologic examination of the heart in our patient; therefore, the precise cause of death is unclear. The contribution of iron deposition to the pathogenesis of late-onset CACTD warrants further study, as it may provide insight into potential treatment strategies for late-onset CACTD.

## Data Availability Statement

The datasets presented in this study can be found in online repositories. The names of the repository/repositories and accession number(s) can be found in the article/supplementary material.

## Ethics Statement

The studies involving human participants were reviewed and approved by the Ethics Committee of the Sixth Affiliated Hospital of Sun Yat-sen University (approval number: 2017ZSLYEC-105). Written informed consent to participate in this study was provided by the participants' legal guardian/next of kin.

## Author Contributions

All authors listed have made a substantial, direct and intellectual contribution to the work, and approved it for publication.

## Conflict of Interest

The authors declare that the research was conducted in the absence of any commercial or financial relationships that could be construed as a potential conflict of interest.
